# Exploring the EEG mu rhythm associated with observation and execution of a goal-directed action in 14-month-old preterm infants

**DOI:** 10.1038/s41598-019-45495-3

**Published:** 2019-06-20

**Authors:** Rosario Montirosso, Caterina Piazza, Lorenzo Giusti, Livio Provenzi, Pier Francesco Ferrari, Gianluigi Reni, Renato Borgatti

**Affiliations:** 1 Scientific Institute, IRCCS “E. Medea”, 0-3 Center for the at-Risk Infant, Bosisio Parini, Lecco Italy; 2 Scientific Institute, IRCCS “E. Medea”, Bioengineering Laboratory, Bosisio Parini, Lecco Italy; 3 CNRS/Université Claude Bernard, Institut des Sciences Cognitives Marc Jeannerod, Lyon, France; 4 Scientific Institute, IRCCS “E. Medea”, Neuropsychiatry and Neurorehabilitation Unit, Bosisio Parini, Lecco Italy

**Keywords:** Social neuroscience, Human behaviour

## Abstract

Electroencephalographic mu rhythm desynchronization is thought to reflect Mirror Neuron System (MNS) activity and represents an important neural correlate of the coupling between action execution and perception. It is still unclear if the MNS in human ontogeny is already available at the beginning of postnatal life and how early experience impacts its development. Premature birth provides a “natural condition” for investigating the effects of early, atypical extra-uterine experience on MNS. The main aim of the present study was to investigate whether the MNS activity is associated with prematurity. We compared the mu rhythm activity in preterm (PT) and full-term (FT) 14-month old infants during an action observation/execution (AO/AE) task. Mu rhythm desynchronization was computed over frontal, central, parietal and occipital regions. Both groups showed mu rhythm suppression in all the scalp regions during action execution. Different desynchronization patterns emerged during action observation. Specifically, FT infants showed mu suppression in the right frontal, bilateral parietal and occipital regions; whereas PT infants exhibited mu suppression only in the right parietal region. Overall, these preliminary findings indicate that an atypical extra uterine experience might have an impact on the MNS activity.

## Introduction

Accumulating evidence suggests that young infants rely on their motor system when they observe someone else performing actions^1–3^. Several studies suggested that infants are provided with a mirror mechanism, that enable them to create, at the cortical level, a bidirectional representation of both action perception and their own action execution^4,5^. The mirror neuron system (MNS), which activates itself during both the execution and the observation of goal directed actions, might represent an important neural correlate of this action-perception coupling. It has been proposed that the MNS relies on mapping of an observed action onto one’s own motor representation^6^. The sensorimotor mapping would provide the observer’s embodied access to the meaning of the observed action. Therefore, the motor system is central to both controlling the bodily movements and supporting cognitive functions associated with the decode processes of others’ actions^7^.

The electroencephalographic (EEG) mu rhythm is a sensorimotor rhythm which is characterized by frequencies that fall in the range of the alpha rhythm and has been identified both in the premotor/motor areas and in the parietal regions^8^. The mu-rhythm has been associated with the activity of the MNS. Recent neurophysiological experiments in the monkey have confirmed this hypothesis by using simultaneous recording of mirror neurons activity from premotor area F5 and EEG from the scalp, and finding a clear inverse correlation between the activity of mirror neurons (calculated through local field potentials) and the amplitude of EEG mu rhythm suppression over central electrodes^9^. The mu rhythm has been used as a neural marker of the MNS activity in human adults and infants^8,10–15^. Indeed, its activity has been found to be suppressed both when an infant executes an action and when he/she observes someone else executing the same goal-directed action^16–18^. For example, in a recent EEG study, exploring the mu rhythm in 7-month-old infants, event-related desynchronization at central sites during action observation was observed when infants subsequently reproduced the experimenter’s goal (i.e., grasping an object^12^.

While these findings suggest that the mirror mechanism gradually emerges during early development and that the mu desynchronization is associated with the activation of the motor system, the origin and the development of this mechanism is still debated^7,19^. Specifically, if the MNS in human ontogeny is already available at the beginning of postnatal life and how early experience impacts its development remain open questions^7,20–24^. Studies aimed to inquiry EEG mu suppression to execution or observation of actions in infant with atypical early experience might therefore shed light on this issue.

Preterm birth represents a major health care issue associated with greater risk for developing social and cognitive difficulties, including imitative behavior^25^. In Neonatal Intensive Care Unit (NICU) hospitalization, during a critical period for infant brain development, preterm infants are exposed to numerous stressors, including painful stimuli, disruption of sleep, excessive physical stimulations. Moreover, NICU staying implies a mother-newborn separation, resulting in a disruption of the normally occurring physical contact and emotional closeness between the mother and infant^26^. Importantly, even in the absence of significant clinical conditions, early adversity which involves altered sensory stimulations and limited contacts with the caregivers may contribute to infants’ socio-emotional difficulties later in life^27^ and have an impact on brain development^28^. Thus, premature birth provides a “natural condition” for investigating the effects of early, atypical extra-uterine experience on MNS associated with the action-perception system. On the one hand, if the MNS is the result of a strong developmental canalization of the brain, highly determined by genetic factors, then the early extra-uterine experience of preterm children would have just a limited impact on the neuroanatomical network supporting the activation of the MNS during execution or observation of actions. On the other hand, if the mirror mechanism is part of an experience-dependent brain network, its development should be affected by early sensorimotor experiences. It follows that early adverse extra-uterine experiences would be likely associated to atypical activity of the MNS. These two hypotheses are not necessarily mutually exclusive, as others have reported^7^, since it is likely that most of brain networks build through complex interactions with the environment and their construction undergoes changes that are somehow canalized but nevertheless still modifiable by early experience.

To explore whether the MNS activity was associated with the prematurity, we investigated the mu rhythm response in infants born full-term and preterm during an action observation/reproduction task at 14 months of age (corrected for prematurity). We recorded EEG mu rhythm while infants observed an experimenter carrying out a goal-directed action (i.e., a button press) that they suddenly were asked to reproduce^2^. Importantly, previous studies suggest that MNS activity might be more evident before the observed action culmination once infants could anticipate its occurrence^17^. Accordingly, in the current study, we focused on the mu rhythm activity which occurred prior of action observation culmination.

As the association between mu rhythm desynchronization and prematurity has not been investigated in previous literature, no specific hypothesis was advanced about how the MNS might be affected by birth status. However, given that the sensorimotor experience has a critical role in the construction of the mirror networks^24^, we expected that preterm infant could exhibited some perturbations in the MNS activity. This hypothesis was grounded on a recent MRI study which documented alterations in connectivity in networks related to motor, language and cognitive function, even in absence of anatomical imaging evidence of injury^29^. Given that networks examined are associated with sensorimotor and cognitive neural systems known to reflect an alteration in maturation in preterm infants, we anticipated that alterations in connectivity could also have an impact on the networks maturation related to MNS activity in children born premature.

## Methods

### Participants

Twenty-seven preterm (PT) infants (15 females) were enrolled at the Neonatal Intensive Care Units (NICUs) of Valduce hospital (Como, Italy) and Manzoni hospital (Lecco, Italy). Inclusion criteria were: gestational age <37 weeks, no documented neurologic disorders as shown by cerebral ultrasound (periventricular leukomalacia up to stage 1; intraventricular hemorrhage up to stage 1 or 2), no sensory deficits (retinopathy up to stage 1 or 2), neonatal hearing screening (ABR or otoemissions) within the norm at the 34^th^ week, and no malformative syndromes and/or major malformations.

A group of thirty-three full-term (FT) infants (gestational age ≥37 weeks and birth weight ≥2500 g, 18 females) was recruited at the Pediatric Unit, Sacra Famiglia hospital (Erba, Italy). Full-term infants had no pathologies or prenatal/perinatal risk factors at birth.

Parents who gave their willingness to participate to the study were contacted by an assistant researcher when their child was 13 months of age (corrected for prematurity). Infants were then tested at 14 months of age using an action observation/execution task while EEG data were recorded. Written informed consent was obtained from all parents prior to testing. The study protocol was approved by the Ethics Committee of the Scientific Institute, IRCCS Eugenio Medea (Bosisio Parini, LC, Italy). All the procedure of the study was conducted in accordance with the Helsinki Declaration.

Twenty infants (PT = 8, FT = 12) were excluded because of technical problems (FT = 3), intolerance to the EEG cap (PT = 2, FT = 1), absence of imitative behaviors (PT = 3, FT = 3), distress during the task (PT = 2, FT = 1) or insufficient amount of good EEG data available (PT = 1, FT = 4). Thus, the final subject sample was made of twenty-one FT and nineteen PT infants.

### Procedure

Perinatal variables, socio-demographic characteristics, fine motor development information and EEG data were collected for both groups of infants. Infants’ perinatal variables including gestational age and length of stay in the hospital/NICU were obtained from medical records. Socio-demographic data such as maternal age, years of education and socio-economic status were obtained for both parents through a questionnaire. The socio-economic status was scored according to the Hollingshead classification^30^ whereby a score ranging 0–90 (0 = occupations that do not require high school graduation; 90 = occupations that require highly specialized education and training) was assigned to each parental job and the higher of two scores was used when both parents were employed.

Due to possible differences in the fine motor skills requested for performing imitation at 14 months of age, infants were tested on the fine motor subscale of the Bayley Scales of Infant and Toddler Development, Third Edition^31^. The test was performed by an assistant researcher at the infant’s home.

EEG data were acquired for each participant during an action observation and an action execution task. Specifically, the protocol consisted of a sequence of trials, each composed by three parts: a baseline (BL) period, an action observation (AO) epoch and an action execution (AE) epoch. The action performed was a button press. According to previous studies^32^, a wood-box with a recessed button that produced an animal sound (e.g., dog barking) was custom-made. The box was connected to the EEG system so that the button press sent a trigger to the recorded EEG signals. At the beginning of each trial, before the BL period, the experimenter hid the button-box under the table and pressed the button. In this case the button press did not produce any sounds but indicated the beginning of the BL epoch, lasting two seconds. During this time no stimuli were presented to the infant, who was expected to be quiet, looking at the experimenter and waiting for his action, thus resting EEG activity was registered. Successively, the experimenter brought out the button box and the infant observed him carrying out the action of pressing the button with the right index finger (AO epoch). Finally, during the AE condition the infant was asked to perform the same action previously observed. The use of animal sounds aimed only at make the procedure more engaging for the infants. The experiment continued as long as the infant remained collaborative, interested and tolerated the EEG cap. On average 24.7 (SD = 7.8, range = 12–37) and 23.7 (SD = 4.3, range = 17–33) trials were performed by the FT and PT group, respectively. No differences between groups emerged in the number of trial accomplished, *t*(31.6) = −0.502; *p* = 0.619. The task was videotaped with a digital camera (Sony HDR-HC9, HDV 1080i) connected to the EEG system, so that the video was synchronous with the EEG recorded data. Moreover, the video recording was used to reject trial epochs in which the infant was not behaving according to the task. In particular, we excluded: BL epochs in which the infant was not quiet; AO epoch in which the infant was not paying attention to the experimenter gesture, performed gross motor movements or movements similar to a reaching, pointing or button-pressing; AE epoch in which the infant didn’t appropriately reproduce the button press by imitation. As a result, a mean of 17.7 BL (SD = 7.1, range = 8–35), 18.5 AO (SD = 6.9, range = 10–34) and 18.4 AE (SD = 6.7, range = 7–31) epochs remained for the FT group and a mean of 14.7 BL (SD = 3.8, range = 9–23), 19.2 AO (SD = 4.4, range = 13–29) and 18.5 AE (SD = 4.5, range = 10–29) epochs remained for the PT group. No differences between groups emerged in the number of epochs selected (*t*-test for BL epochs: *t*(31.485) = −1.679, *p* = 0.103; *t*-test for AO epochs: *t*(38) = 0.396, *p* = 0.694; *t*-test for AE epochs: t(35.117) = −0.082, *p* = 0.935).

The video recording was used also to check the hand used for performing the execution task. For each infant a lateralized manipulation score^33^ was computed in order to verify the hand preference. The score considers the percentage of left- and right-hand use during the button press task. Specifically, the percentage of left-hand use was subtracted from the percentage of right-hand use, and this was divided by the square root of the sum of percentage right- and left-hand use: (%R − %L)/SQRT(%R + %L). This results in a score between 1 and −1, where positive values indicate a right preference in hand use whereas negative values indicate a left preference.

### EEG data acquisition and processing

EEG data were acquired at the IRCCS E. Medea Baby Lab. At their arrival to institute, the infant with his/her mother was accompanied by the experimenter into a play-room next to the EEG laboratory. There, the experimenter played with the infant for approximately 15 minutes letting him/her to familiarize with the lab environment and with the unfamiliar adult. When the infant felt at ease he/she was prepared for the EEG acquisition. EEG signals were acquired in a sound-attenuated and electrically-shielded room while the infant sat at a table on his/her mother lap, facing the experimenter. EEG data were recorded with a 60/64-electrode cap (HydroCel Geodesic Sensor net, Electric Geodesic, Inc., Eugene, Oregon), using the vertex as reference, 250 Hz sampling rate and 0.1–100 Hz online bandpass filtering. After recording, EEG data were exported to a Matlab (The Mathworks Inc., 2016) compatible format and processed within the EEGLAB signal processing toolbox^34^ and custom Matlab® scripts. Continuous EEG data were filtered using a 1-Hz high pass and then a 45-Hz low pass FIR filter. Channels with high impedance (>50 KΩ) or visually evident noise were interpolated with a spherical spline. No more than 15% of channels out of 60 were interpolated (M = 4.2, SD = 1.9, range = 0–8). The EEG signals were then re-referenced to average reference. EEG data were successively segmented according to the behavioral events, which were automatically triggered by the button press. Specifically, BL epochs of 2000 ms and AO/AE epochs of 1500 ms were considered. For the latter epochs we used the 1500 ms prior to the experimenter or infant button press, which is approximately the timing from the beginning of the arm movement to action culmination^16^. Epochs containing artifacts were identified and rejected using an automatic algorithm (abnormal amplitude test with threshold 200 μV)^35^ followed by visual inspection. As a result, a mean of 12.0 BL (SD = 4.4, range = 5–19), 12.3 AO (SD = 5.7, range = 5–27) and 10.8 AE (SD = 4.3, range = 5–18) epochs remained for the FT group and a mean of 10.5 BL (SD = 3.8, range = 5–17), 12.5 AO (SD = 3.8, range = 5–18) and 10.7 AE (SD = 3.7, range = 5–18) epochs remained for the PT group. No differences between groups emerged in the number of epochs selected (*t*-test for BL epochs: *t*(38) = −1.006, *p* = 0.321; *t*-test for AO epochs: *t*(38) = 0.156, *p* = 0.877; *t*-test for AE epochs: t(38) = −0.060, *p* = 0.952).

For each considered electrode and epoch the power spectral density (PSD) was estimated using the Welch’s method (Hamming window of 125 samples and 63 samples overlap) and then averaged across conditions (BL, AO, AE). The PSD was log transformed as follow: PSD = log_10_(1+PSD). Since the lack of agreement in the identification of mu frequency bands in infancy^36^ we decided to use individualized frequency bands, as previously done in infant-mu-rhythm literature^16,17,37,38^. To determine the band of interest we calculated each infant’s maximally attenuated frequency during the AE phase in respect to the BL and we selected a 3 Hz band centered on this frequency. The identification of the maximally attenuated frequency was done using a broad frequency band (4–13 Hz) and averaging the identified value across channels^39^. To ensure the weak stationarity of the analyzed epochs, an outlier analysis was performed. Specifically, all the epochs for each subject and each condition with a power in the identified frequency band that was higher or lower than 3 standard deviation across trials were excluded for further analysis. Only two epochs were rejected in this step of the analysis: an AO epoch in the FT group and an AE epoch in the FT group. Finally, event-related changes in band power between the BL and AO, AE epochs were calculated as follow: event-related desynchronization\synchronization (ERD\ERS) = [(A − R)/R]*100^40^; where A denotes the average power during the AO or AE epochs and R denotes the average power during the BL epochs. ERD/ERS indicates either suppression or enhancement of EEG power relative to a baseline level.

**Figure 1:**
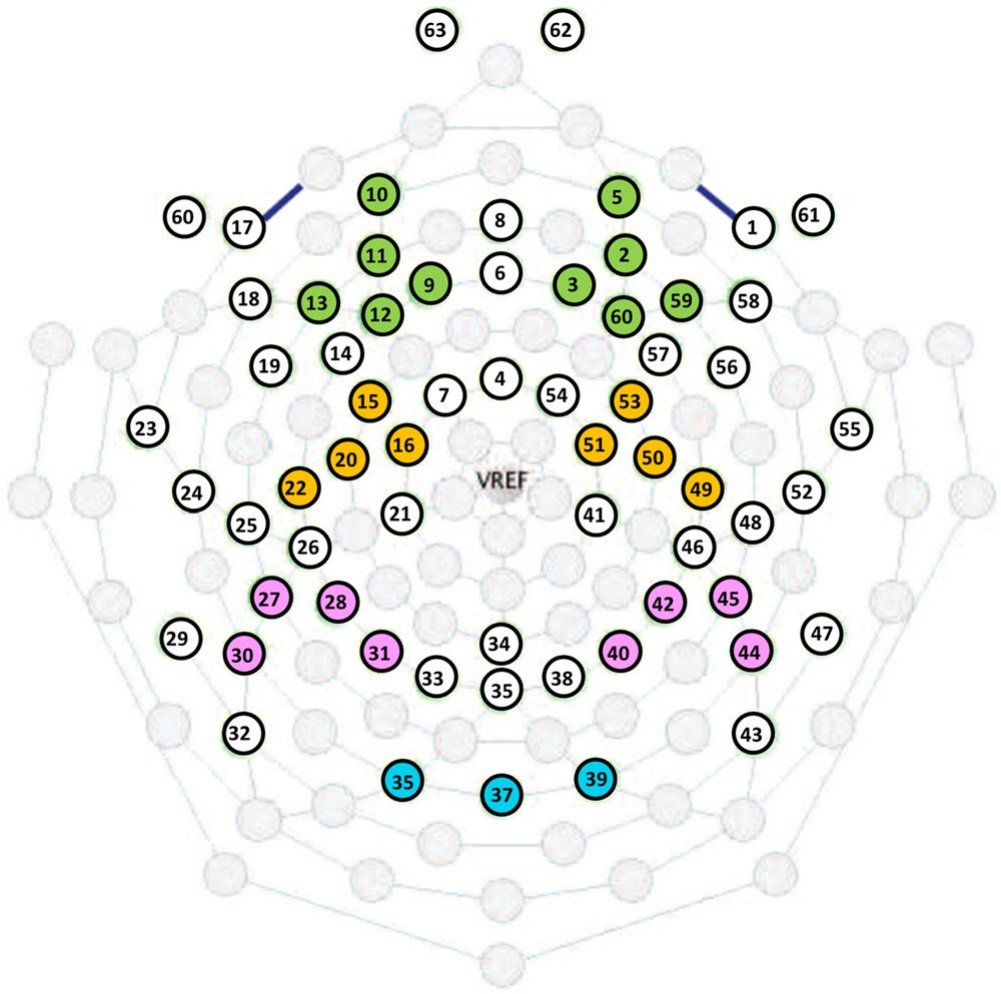
Sensor layout of the 60/64 channel Hydro-Cel Geodesic Sensor Net used in the study. Electrode clusters are represented as follow: frontal in green, central in yellow, parietal in pink and occipital in light-blue.

As suggested in previous reviews^32,36^, event related power changes were analyzed in multiple locations across the scalp topography (i.e., frontal, central, parietal and occipital regions). Specifically, the following clusters were created by averaging the single channel ERD/ERS values: frontal left (F_left), electrodes: 9, 10, 11, 12, 13 (corresponding respectively to sites F1, FP1, AF3, F3, F5 of the 10–10 system); frontal right (F_right), electrodes: 2, 3, 5, 59, 60 (corresponding respectively to sites AF4, F2, FP2, F4, F6 of the 10–10 system); central left (C_left), electrodes: 15, 16, 20, 22 (corresponding respectively to sites FC3, C1, C3, C5 of the 10–10 system); central right, (C_right), electrodes: 49, 50, 51, 53 (corresponding respectively to sites C6, C4, C2, FC4 of the 10–10 system); parietal left (P_left), electrodes: 27, 28, 30, 31 (corresponding respectively to sites P5, P3, P7, P1 of the 10–10 system); parietal right (P_right), electrodes: 40, 42, 44, 45 (corresponding respectively to sites P2, P4, P8, P6 of the 10–10 system) and occipital (O), electrodes 35, 37, 39 (corresponding respectively to sites O1, Oz, O2 of the 10–10 system). See Fig. 1.

### Statistical analysis

Preliminarily statistical analyses evaluated perinatal variables, socio-demographic characteristics and fine-motor scores. Inter-groups differences were analyzed through a series of independent samples *t*-tests.

First, a repeated-measures ANOVA was performed including the within-subject factor *cluster* (7 levels: F_left, F_right, C_left, C_right, P_left, P_right and O) and the between-subject factor *group* (2 levels: FT *vs*. PT), separately for AO and AE condition. Greenhouse-Geisser correction was applied when appropriate. Bonferroni correction for multiple comparisons was applied in post-hoc analysis.

Secondly, significant mu rhythm desynchronization during AO and AE was tested for each cluster^36^. This was done by contrasting ERD/ERS values against a null hypothesis of no change in power^32,41^ using a series of one-sample *t*-test, separately for the PT group and the FT one.

A series of correlations were performed in order to analyze if mu rhythm suppression in frontal, central, and parietal electrodes was related to occipital alpha activity. Furthermore, a series of additional correlations were performed to explore possible associations between perinatal variables (i.e., gestational age, length of stay in the hospital) and mu rhythm activity. In both analysis Pearson correlations were calculated separately for the two groups and taking into account only the clusters that showed a significant desynchronization.

All analyses were performed at a significance level *p* ≤ 0.05, using SPSS for Windows (version 21, Chicago, IL, USA).

## Results

### Perinatal, socio-demographic and motor characteristics

As reported in Table 1 significant differences emerged between FT and PT for perinatal variables. No statistical differences were found for socio-demographic variables (i.e., mother’s age and family SES) and the Bayley fine motor score. The latter suggested that fine motor abilities were in the normal range for preterm infants, thus they should not influence their imitation performance. Indeed, as previously reported we didn’t find any difference between FT and PT infants in the number of successful AE epochs. Consistent results were found for the number of unsuccessful AE epochs. On average the FT infants didn’t adequately reproduce the observed action in 6.3 trails (SD = 4.8, range = 0–17), while the PT infants were not able to correctly imitate in 5.1 trials (SD = 3.2, range = 1–14), *t*(35.129) = −0.926, *p* = 0.361). Moreover, the imitation task was rapidly understood by both groups of infants. To asses this issue, we have considered the infant behavior at the beginning of the procedure (we arbitrarily used the first five trials) like a measure of the comprehension of the imitation task. Specifically, we counted the number of unsuccessful AE epochs in the first five trials. No differences between groups emerged (FT group: mean = 1.5, SD = 1.6, range = 0–5; FT group: mean = 0.8, SD = 1.0, range = 0–3; *t*(38) = −1.522, *p* = 0.136).

**Table 1:** Infants and family characteristics of the two groups.

	Full-term Group (N = 21)		Preterm Group (N = 19)		Unpaired *t*-test	
	*Mean*	*SD*	*Mean*	*SD*	*t*	*p*
**Perinatal Variables**						
Gestational age (weeks)	39.80	0.95	30.32	7.54	17.69	<0.001
Length of stay in hospital (days)	2.90	0.90	33.64	10.87	−9.36	<0.001
**Socio-Demographic Characteristics**						
Socioeconomic status (score)*	57.08	14.10	45.91	20.95	1.72	0.10
Mother’s age (years)	33.78	3.95	34.45	4.25	−0.44	0.67
**Infants Characteristics at Examination**						
Age (months)**	13.83	0.25	13.95	1.65	−0.27	0.78
Bayley fine motor score	28.31	2.02	27.86	1.83	0.64	0.53

*Hollingshead’s (1975) classification.

**Corrected age for prematurity.

The imitation performance was also analyzed in relation to the infant hand preference in performing the execution task. Among the 40 infants tested in the study only 17 performed the button press task always with the same hand (13 infants (7 FT and 5 PT) used the right hand and 4 infants (3 FT and 1 PT) used the left hand). Nevertheless, a hand preference was mostly present. Table 2 summarizes the results of the lateralized manipulation score analysis. Specifically, for each group the number of infants that had a right or left hand preference and the respective lateralized manipulation scores (median values and interquartile ranges) are shown. In both groups, most of the infants had a right hand preference in performing the task. No differences, between groups emerged in relation to the score, thus suggesting no difference in hand use (Mann-Whitney test: Z = −0.924, *p* = 0.356).

**Table 2:** Infant hand preference characteristics.

	Full-term Group			Preterm Group		
	N	Median	IQR	N	Median	IQR
Right-hand preference	14	0,92	0,47	16	0,89	0,30
Left-hand preference	7	−0,90	0,27	3	−0,81	0,25

Number of infants with right and left preference (i.e. positive and negative values of the lateralized manipulation score respectively) and median values, inter quartile ranges (IQR) of the lateralized manipulation score are shown.

### Mu rhythm range

**Figure 2:**
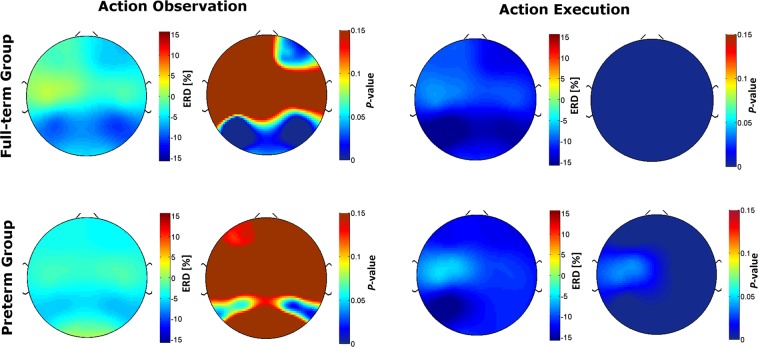
Topographic maps of mu rhythm desynchronization (ERD [%]) and respective topographic maps of the one-sample *t*-test *p*-values (significant values are shown in blue).

The mean frequency with the maximal PSD suppression during AE was 6.6 Hz (SD = 1.0, range = 5–9) for the FT group and 6.7 Hz (SD = 1.2, range = 5–8) for the PT group with no differences between groups *t*-test: *t*(38) = 0.336; *p* = 0.739. This led to the identification of bands of analysis ranging 4–10 Hz, which are in line with previously reported frequencies of the mu rhythm in infancy^13,17,32,42^. ERD/ERS values for each condition and clusters are reported in Table 3 and shown in Fig. 2.

**Table 3:** ERD/ERS values for each channel clusters and results of the one-sample *t*-test used to investigate the strength of the desynchronization against a null hypothesis of no change in power during action observation (AO) epoch and action execution (AE).

	AO				AE			
	*Mean*	*SD*	*One sample t-test*		*Mean*	*SD*	*One sample t-test*	
			*t*	*p*			*t*	*p*
**Full-term Group**								
F_left	−1.94	10.06	−0.88	0.388	−9.43	7.40	−3.90	**<0.001**
F_right	−5.49	11.98	−2.10	**0.048**	−12.62	8.63	−6.46	**<0.001**
C_left	0.62	11.29	−0.25	0.803	−8.34	8.84	−4.48	**<0.001**
C_right	−2.45	9.38	−1.18	0.246	−9.89	7.09	−5.61	**<0.001**
P_left	−8.62	10.86	−3.63	**0.002**	−17.00	7.80	−9.60	**<0.001**
P_right	−9.84	9.49	−4.75	**<0.001**	−14.86	8.62	−7.32	**<0.001**
O	−5.40	10.90	−2.27	**0.034**	−11.29	11.21	−4.96	**<0.001**
**Preterm Group**								
F_left	−3.69	10.41	−1.54	0.140	−12.03	8.21	−4.49	**<0.001**
F_right	−4.18	15.60	−1.17	0.258	−12.49	9.49	−4.69	**<0.001**
C_left	−2.50	11.37	−0.96	0.350	−5.98	11.47	−3.54	**0.036**
C_right	−2.16	12.35	−0.76	0.456	−10.36	11.46	−4.66	**0.001**
P_left	−5.60	13.01	−1.88	0.077	−15.88	11.44	−7.52	**<0.001**
P_right	−6.01	12.20	−2.15	**0.046**	−11.22	10.93	−5.61	**<0.001**
O	0.53	13.77	0.17	0.869	−11.32	10.69	−4.06	**<0.001**

Significant values in bold.

### Action observation

Repeated measure ANOVA showed no main effect of *group* and of *cluster* X *group* interaction effect. A significant main effect of *cluster* was found, *F*(6, 228) = 4.45, *p* < 0.001. From the post-hoc analysis emerged that: the P_right cluster (M = −7.93, SD = 1.72) showed greater desynchronization then the F_left cluster (M = −2.81, SD = 1.62) (*p* = 0.028), the C_left cluster (M = −0.94, SD = 1.79) (*p* = 0.001) and the C_right cluster (M = −2.30, SD = 1.72) (p = 0.015). Whereas, the P_left (M = −7.12, SD = 1.89) cluster presented greater desynchronization than the C_left cluster (*p* 0.047). Thus, a similar mu rhythm desynchronization emerged between the parietal regions and the frontal right region.

In FT group, one-sample *t*-tests (Table 3, Fig. 2) indicated that mean ERD/EDS values were significantly different from zero for frontal right *t*(20) = −2.10, *p* = 0.048, parietal left *t*(20) = −3.64, *p* = 0.002, parietal right *t*(20) = −4.75, *p* < 0.001 and occipital *t*(20) = −2.27, *p* = 0.034 regions, but not for the central regions. In the PT group a significant desynchronization was found only in the right parietal cluster, *t*(18) = − 0.76, *p* = 0.046.

### Action execution

Repeated measure ANOVA showed no main effect of *group* and of *cluster* X *group* interaction effect. The *cluster* main effect was significant, *F*(6, 228) = 5.49, *p* < 0.001. Follow-up contrasts showed greater desynchronization over the parietal regions than over the centrals. Specifically, the F_right cluster (M = −12.56, SD = 1.46) showed greater desynchronization then the C_left cluster (M = −7.16, SD = 1.59) (p = 0.015); the P_left cluster (M = −16.44, SD = 1.56) showed greater desynchronization than the C_left cluster (*p* < 0.001) and the C_right cluster (M = −10.13, SD = 1.56; *p* = 0.003). Finally, the P_right cluster (M = −13.04, SD = 1.60) showed greater desynchronization than the C_left cluster (*p* = 0.003).

One-sample *t*-tests indicated that mean ERD/EDS values were significantly different from zero for all regions both for FT and PT group (all *p*s ≤ 0.036, see Table 3 and Fig. 2 for detailed results).

### Correlation analysis

During the AO, in full-term group, the suppression measured over the occipital site was significantly correlated with both parietal suppression (P_left cluster: *r* = 0.618, *p* = 0.003; P_right cluster: *r* = 0.703, *p* = 0.000) and F_right cluster suppression (*r* = 0.619, *p* = 0.003). In preterm group, no significant association emerged between clusters that showed a significant desynchronization (P_right cluster) and the occipital electrode activity. During the AE, in full-term group, although mu suppression was significantly different from zero on frontal, central, and parietal clusters, the suppression measured over the occipital site was significantly correlated only with P_right cluster (*r* = 0.585, *p* = 0.005) and C_left cluster suppression (*r* = 0.450, *p* = 0.041). In preterm group, no significant associations emerged between brain areas that showed a significant desynchronization (i.e., all clusters) and the occipital electrode activity.

No significant associations emerged between perinatal variables and mu rhythm desynchronization.

## Discussion

The main aim of the current study was to explore if an atypical development condition (i.e., prematurity) could have an impact on EEG mu rhythm desynchronization during observation and execution of a goal-directed action (i.e., button press). The mu rhythm activity was: 1. compared between the two groups and, 2. analyzed against the null hypothesis of no change in power, for PT infants and FT infants separately.

Preliminarily, it should be noted the frequency bands analyzed for the studying of the mu rhythm activity. Previous research has documented mu rhythm attenuation, commonly measured in the predefined frequency band 6–9 Hz, when infants observe a goal-directed action and perform the same action^13,32^. Consistently, using an individualized frequency bands approach^17,42^, we identified a maximally attenuated frequency during the action execution phase around 6.5 Hz.

Contrary to our expectation, we did not find any significant difference in the mu desynchronization between preterm and full-term group when infants observed the goal-directed action and performed the same action. Moreover, correlational analysis showed that factors associated with prematurity, such as gestational age and the length of stay in the hospital were unrelated with mu desynchronization over brain regions. Overall, these findings seem to suggest that mirror mechanisms associated with action-perception system were unaffected by birth status and that early extra-uterine experience of preterm children would have no impact on the MNS activity. Nevertheless, analyses against the null hypothesis of no change in power showed different desynchronization patterns in the two groups. While during action execution both groups showed a broad significant attenuation of the mu rhythm; during action observation full-term infants showed a significant mu desynchronization in right frontal and bilateral parietal regions, whereas preterm infants showed significant mu suppression only in the right parietal region.

As above mentioned during action execution, mu desynchronization was significantly different from zero over frontal, central and parietal clusters in both preterm and full-term infants. While mu suppression during action execution is usually reported over central electrodes^32,43^, some researchers have suggested that in infancy the fronto-parietal activities may be also engaged in this condition^16,17^.

As previously reported, during action observation, infants showed a significant mu rhythm desynchronization in both frontal (only in the full-term group) and parietal regions, but unexpectedly not in the central one. Functional neuroimaging studies identified three main cortical areas that correspond to motor function and are activated during action observation in humans: the premotor cortex; the posterior part of the inferior frontal gyrus; and the inferior parietal lobe^44^. Interestingly, more recent studies and anatomical analyses of the monkey mirror network, have shown that other regions, beyond the traditional parietal-premotor network have mirror-like activities or could be part of a grasping extended network^45,46^. Such cortical regions include the ventrolateral prefrontal cortex and pre-supplementary cortex. Thus, during the execution and observation of hand actions several cortical areas, beyond the parietal-premotor network, could be simultaneously active. In line with this, other studies showed that in action-observation tasks prefrontal regions could be involved, for example when the adult participants had to observe actions from different perspectives^47,48^. This and other studies seem to point out a possible involvement of prefrontal regions for actions to be copied^4^ or that might require spatial processing of visuomotor information typically occurring in imitative tasks. Notably, infant mu desynchronization to action observation has been reported in several scalp locations, over frontal, central and parietal regions^32,37,49^, but parietal regions and premotor cortex are considered the most likely source of mu suppression during action observation^8,50^. A possible explanation for the differences in mu desynchronization between other studies and ours, might be related to a number of variables related to procedural and methodological aspects, such as electrodes included in the cluster analysis and EEG epochs took in consideration. Particularly, most investigations considered EEG epochs that included both periods preceding and successive the action, here we considered the 1500 ms before the experimenter or infant button press. The emphasis on mu suppression prior the culmination of the observed action (i.e., button press) was based on the hypothesis that EEG desynchronization is particularly strong when infants anticipate the grasping occurrence few millisecond before the observed hand touched the object, namely during the reaching phase^16^. The presence in our result of a frontal-parietal desynchronization confirm that 14-month-old infants activated MNS when they can foresee the experimenter executing the hand goal-directed action, providing further evidence that MNS activity during action observation may also reflect a process of anticipating how an action will unfold^51,52^.

Importantly, during action observation, in full-term infants our results suggest a major contribution of the right hemisphere in the mu rhythm desynchronization. This is partially inconsistent with previous human mu rhythm studies. In fact, both adult^53–55^ and infant studies that analyzed mu rhythm activity are inconclusive regarding lateralization effects. In infant research, mu rhythm attenuation during action observation has been reported bilaterally^32^, stronger in the left hemisphere^16^ or right lateralized when infants at 9 months observe facial expressions^10^.

In the preterm group, during action observation we observed significant mu-rhythm suppression only in the right parietal electrodes, differently from the FT group where the EEG suppression was bilateral. This may suggest a less-than-optimal MNS system activation when these infants were observing the action. Interestingly, this finding is reminiscent of results reported in atypically developing individual, such as autistic spectrum disorders (ASD) and Down Syndrome (DS). An abnormal mu suppression has been documented when ASD individuals viewed actions performed by others, despite normal suppression when they performed the same actions, suggesting an execution/observation matching system dysfunction in individuals with autism^56,57^. Using magnetoencephalography, one study reported that adults with DS showed a significantly reduced overall attenuation of mu rhythm when they viewed the movements made by the experimenter^41^. Although there is a plethora of key distinctions among ASD, DS and prematurity, overall this evidence suggests that there is either a dysfunction or maturational delay in the observation/execution matching system in population with atypical development suggesting that the mu rhythm might be a marker of altered neural development, with possible functional involvement of the MNS.

During both observation and action execution full-term infants showed a significant alpha range desynchronization also in the occipital region. Similar activation was found in preterm infants only during the action execution. Importantly, in full-term infants, but not in preterm infants, occipital activity was significantly correlated with both parietal and frontal mu suppression during the action observation. This finding raises the question of the possible interconnection of mu rhythm and occipital alpha activity associated with visual attention in observation conditions^58–60^. Indeed, a previous study suggests that occipital alpha suppression may be associated with attentional processes^61^, so that in full-term infant, the parietal/frontal mu rhythm attenuation during action observation could have been driven by both mirroring and attentional processes^12^.

Actually, only few mu rhythm studies have reported occipital alpha in order to ensure that central-parietal electrodes activities were independent from alpha rhythm in occipital region^44,58^. An occipital activation during different imitation tasks was previously reported, suggesting an association in alpha desynchronization between cortical sites (e.g.^12^). In an action observation/execution task a central-parietal suppression was significantly correlated with occipital alpha suppression^38^. On the other hand, a recent study using connectivity analyses has reported that central mu and occipital alpha are distinct, yet correlated, foci of activity, suggesting that mu desynchronization could be a robust index of infant mirroring system activity^58^. In sum, given that literature findings are still debating about the contribution of the occipital desynchronization to the mu rhythm, we cannot exclude that, particularly in the action observation, the suppression in the alpha frequency band could be indicative of attentional processes rather than mirroring. According with previous view, the occipital suppression might suggest that in this condition, infants payed extra attention to the presentation of actions they are learning^38^.

While we acknowledge that current findings do not offer direct evidence regarding the role of connectivity in networks related to MNS activity, our findings lead us to the notion that preterm birth could be associated with a less-than-optimal networks maturation, where MNS could be one of the key hubs involved in action-perception links, which are particularly relevant in imitation tasks. This hypothesis remains speculative; however, the evidence documenting network connectivity alterations of sensorimotor and cognitive neural systems in preterm infants^38^, supports this view. Further research is needed to disentangle which factors associated with prematurity could be related with this alteration, such as amount of NICU-related stress, mother-newborn separation, etc. and how this potential alteration could be related to socio-cognitive competences, such as imitation skill.

Interestingly, an EEG study on newborn macaques aged three days, showed that nursery-reared macaques have a weaker desynchronization of lower alpha rhythm in the frontal/central electrodes than mother-reared individuals while observing facial gestures^62^. Thus, our data resonate in part with these findings, supporting the view that early adverse experience may lead to dysfunctional or hypo-functional activity in the MNS. Moreover, other data in both human and monkey infants show that the mu rhythm is sensitive to the level of experience an infant has had with an action or its goals^63,64^. Thus, although no difference between preterm infants and full-term infants emerged in the fine motor skills, we cannot rule out the hypothesis that preterm infant’s reactivity to others’ actions could be correlated with a reduced experience in object-directed interactions with the specific experimental task used in the current study.

Lastly, some methodological notes should be highlighted. It is worth noting that the results of the current study do not seem to be ascribed to the potential difference between groups in the basic EEG activity or in motor development. As for this latter, it should be noted that we did not find difference between preterm infants and full-term ones in the fine motor skills. This suggests that dissimilarity in mu suppression pattern, which emerged in the two groups during the motor perception/imitation task cannot be not explained by decreased motor performance of preterm infants. It should be also noted that the motor task was particularly easy for infant at this age. As for EEG activity, a previous electrophysiological research has documented an initial EEG alpha reduction in the preterm group^65^, which in turn suggests a functional alteration in brain development of these infants as a result of prolonged extra-uterine experience and/or prematurity^66^. However, it is unlikely that, at this age, the difference in mu suppression observed in the current study reflects general difference in electrical oscillations between groups due to weaker or altered motor development of the networks controlling hand movements. In addition to this we should also consider that the age of preterm infants was corrected for prematurity, thus the brain maturation is similar to full-term infants. Second, we computed an event-related desynchronization/synchronization index (i.e., ERD/ERS), rather than an absolute power value of the EEG mu rhythm; this procedure reduces the impact of the basic EEG activity on the mu rhythm variations. Third, if the group difference reflected different brain maturation then one would expect to find a difference in the mu rhythm activity across task conditions and scalp locations; actually, we found that mu suppression was condition-dependent. Thus, we could speculate that rather than deficits in the motor programs controlling arm movements, the weak mu rhythm assessed in PT infants might reflect alterations or delays in the strength/maturation of temporo-parieto-frontal connections which brings visual information from temporal regions to parietal-motor regions. Although this remains a hypothesis, further experiments, through structural MRI or DTI imaging, could clarify such important issues.

To the best of our knowledge, this is the first study that explored preterm infants MNS through EEG mu rhythm and some considerations could be pointed out. On the one hand, preterm birth has been associated to social cognition difficulties, such as in the imitation of action sequences^67,68^, in peer play and synchronous interactions with caregivers^69^, in recognizing faces^70^, and in understanding social behaviors^71^. Thus, the atypical EEG activity detected during the action observation may represents one of neural mechanisms which underpin, or simply may be associated to, the preterms’ early social cognition impairments, as well as the long-lasting increased risk of social suboptimal development^72^. On the other hand, these results may provide new insights about the human MNS sensitivity to early environmental adversity as previously theorized^21,62,73^. Previously-cited work on non-human primates by Vanderwert and colleagues^62^ showed that the early postnatal social environment shape behavioral imitation and its neural correlate (i.e. MNS *via* EEG alpha band mu rhythm). In humans, the extent to which mothers mirrored infant facial expressions at two months postpartum predicted infant sensorimotor system activity during observation of the same expressions at nine months^10^. Our finding adds piece of knowledge which suggests that factors associated with prematurity (i.e., early adverse experiences, maternal deprivation) may affect activity of a neural system subserving action perception (i.e., MNS), likely trough the alterations in connectivity in networks related to sensomotory and cognitive functions^29^.

### Limitations of the study

Some limitations in the current study must be acknowledged. The relevant loss of data due to infants’ state (i.e., fussiness and cry) may be considered a procedural bias. Notwithstanding the percentages of missing data were comparable between the two groups and, moreover, we controlled for perinatal and socio-demographic variables while assessing which infants had to be included or excluded from the analysis. Second, although all efforts to control for potential confounding variables were done, we did not rule out that our findings could be associated to other uncontrolled factors which occurred in the preterm infants’ life (i.e., hospitalizations) during the first months of life rather than the birth status *per se*. Thus, further research is needed to corroborate our results. Third, the sample size was relatively small and concerns about statistical power might arise. Thus, the findings generalizability is at the best limited and future studies with larger sample sizes are warranted. Nevertheless, it should be considered that our sample size was in line, or even larger, than those described in previous similar studies^13,16,42,64,74^. Since the sample in this study was solely composed by healthy preterm infants, the generalizability of the findings to all infants born premature is unclear and requires further investigation. Finally, we are conscious of the limitation of scalp EEG analysis. Indeed, scalp recorded EEG is a sum of far-field potentials arising from nearly every active cortical source summed with potentials from non-brain sources and as a consequence there is not a simple relationship between the recorded scalp signals and their cortical sources. Thus, to better investigate the cortical pathways of the MNS in both full-term and preterm infants source localization algorithms that allow the analysis of source resolved signals (e.g.^42,75^ should be performed and the use other imaging techniques (i.e. *f*MRI) are clearly necessary.

## Conclusions

The study of mu rhythm in atypically developing infants could be crucial for the investigation of emerging neural mechanisms underlying the action-perception coupling. Our results have two major implications for our understanding of the ontogeny of the MNS. First, these data provide evidence that in at-risk condition, such as premature birth, the MNS might be altered suggesting that the early extra-uterine sensorimotor experiences may have an impact on the mirror mechanisms. This finding is also in line with previous theoretical view^20,21,73^ and experimental studies in human and nonhuman primates showing that the MNS is sensitive to the quality of early environment experiences^10,62^. Nevertheless, further research is needed to understand how this mechanism operates since the very first days of postnatal life and how it could be affected by different social and non-social experiences, especially under early adverse conditions. Second, our findings suggest that mu desynchronization during action observation may represent a potential marker of the cortical activation, probably including MNS, to inquiry action understanding (i.e., action anticipation) in atypically developing infants in future study. Not only, but based on this approach, the use of action observation and imitation associated with the MNS activity could have an increasing role in the rehabilitation programs in the treatment of children with brain lesions^76–78^.

## Data Availability

The data generated during the current study are available from the corresponding author on request.
